# Cost of TB care and equity in distribution of catastrophic TB care costs across income quintiles in India

**DOI:** 10.1186/s41256-024-00392-9

**Published:** 2024-12-09

**Authors:** Kathiresan Jeyashree, Jeromie W. V. Thangaraj, Devika Shanmugasundaram, Sri Lakshmi Priya Giridharan, Sumit Pandey, Prema Shanmugasundaram, Sabarinathan Ramasamy, Venkateshprabhu Janagaraj, Sivavallinathan Arunachalam, Rahul Sharma, Vaibhav Shah, Bhavani Shankara Bagepally, Joshua Chadwick, Hemant Deepak Shewade, Aniket Chowdhury, Swati Iyer, Raghuram Rao, Sanjay K. Mattoo, Manoj V. Murhekar

**Affiliations:** 1 ICMR-National Institute of Epidemiology (ICMR-NIE), R-127, TNHB, Ayapakkam, Chennai, Tamil Nadu 600077 India; 2 TB Support Network, WHO Country Office for India, New Delhi, India; 3 Central TB Division, New Delhi, India

**Keywords:** Costs of TB care, Catastrophic costs, Direct costs, Indirect costs, India

## Abstract

**Background:**

Tuberculosis (TB) poses a significant social and economic burden to households of persons with TB (PwTB). Despite free diagnosis and care under the National TB Elimination Programme (NTEP), individuals often experience significant out-of-pocket expenditure and lost productivity, causing financial catastrophe. We estimated the costs incurred by the PwTB during TB care and identified the factors associated with the costs.

**Methods:**

In our cross-sectional study, we used multi-stage sampling to select PwTB notified under the NTEP, whose treatment outcome was declared between May 2022 and February 2023. Total patient costs were measured through direct medical, non-medical and indirect costs. Catastrophic costs were defined as expenditure on TB care > 20% of the annual household income. We determined the factors influencing the total cost of TB care using median regression. We plotted concentration curves to depict the equity in distribution of catastrophic costs across income quintiles. We used a cluster-adjusted, generalized model to determine the factors associated with catastrophic costs.

**Results:**

The mean (SD) age of the 1407 PwTB interviewed was 40.8 (16.8) years. Among them, 865 (61.5%) were male, and 786 (55.9%) were economically active. Thirty-four (2.4%) had Drug Resistant TB (DRTB), and 258 (18.3%) had been hospitalized for TB. The median (Interquartile range [IQR] and 95% confidence interval [CI]) of total costs of TB care was US$386.1 (130.8, 876.9). Direct costs accounted for 34% of the total costs, with a median of US$78.4 (43.3, 153.6), while indirect costs had a median of US$279.8 (18.9,699.4). PwTB < 60 years of age (US$446.1; 370.4, 521.8), without health insurance (US$464.2; 386.7, 541.6), and those hospitalized(US$900.4; 700.2, 1100.6) for TB experienced higher median costs. Catastrophic costs, experienced by 45% of PwTB, followed a pro-poor distribution. Hospitalized PwTB (adjusted prevalence ratio [aPR] = 1.9; 1.6, 2.2) and those notified from the private sector (aPR = 1.4; 1.1, 1.8) were more likely to incur catastrophic costs.

**Conclusions:**

PwTB in India incur high costs mainly due to lost productivity and hospitalization. Nearly half of them experience catastrophic costs, especially those from poorer economic quintiles. Enabling early notification of TB, expanding the coverage of health insurance schemes to include PwTB, and implementing TB sensitive strategies to address social determinants of TB may significantly reduce catastrophic costs incurred by PwTB.

**Supplementary Information:**

The online version contains supplementary material available at 10.1186/s41256-024-00392-9.

## Background

Poverty, a key determinant of Tuberculosis (TB) incidence and mortality, is also one of its main consequences [1, 2]. Ending extreme poverty could reduce the global incidence of TB by more than one-third by 2035 [3]. The Sustainable Development Goal 1 to end poverty is closely linked with the End TB goal to achieve zero catastrophic costs. Worldwide, national TB programs offer TB diagnosis and treatment free of cost under the public sector, which are complemented by various social protection strategies that aim to reduce the costs incurred by persons with TB (PwTB).

However, PwTB continue to experience substantial costs of TB diagnosis and treatment [4, 5]. They also face inability to work and loss of productivity which compound their economic challenges and food insecurity 6, 7]. This eventually has adverse impacts on TB transmission and treatment outcomes [7]. Prolonged pathways to care delay the diagnosis, increase the number of visits before treatment initiation, worsen the severity of the disease and diagnosis, increase the chances of unfavorable treatment outcomes [8] and inflate their costs. The average out-of-pocket expenditure (OOPE) incurred by the PwTB in low and middle-income countries (LMICs) during TB care ranges from $1127 to $1417 [9]. Indirect costs contribute more to the total costs than direct costs [9–11]. PwTB resort to coping strategies like dissolving their savings, borrowing money, or selling assets to cope with the substantial expenses incurred for TB care [12]. When these costs exceed 20% of the annual pre-TB household income of the household, the household experiences financial catastrophe [13].

Globally, around 50% of the PwTB experience catastrophic costs due to TB, and high burden countries like India, China, Nigeria, Uganda experience higher costs [14]. Studies conducted in LMICs report high catastrophic costs: 59.1% of households in Egypt [15], 22% in China [16], 60% in Myanmar [17], and 42.4% in Philippines [10]. Catastrophic costs act as a barrier to accessing TB diagnosis [14] and affect treatment adherence and outcomes [18–20]. They hinder the attainment of the End TB aim of achieving zero catastrophic costs for households affected by TB. PwTB who are income earners [18], experience delay in diagnosis [15], have drug-resistant TB (DRTB), or are treated in the private sector [21], are more vulnerable to experiencing higher catastrophic costs [22]. Furthermore, the incidence of catastrophic costs is not equitably borne across income quintiles as TB care costs are unfairly concentrated among the poorer quintiles [23–25].

India, one of the high TB burden countries, has around 10% of its population living below the extreme poverty line [26]. The National TB Elimination Program (NTEP) in India offers TB diagnosis and treatment free of cost. In addition to these, social protection initiatives such as *Ni-kshay Poshan Yojana* (NPY) [27], *Ni-Kshay Mitra*, cash transfers, and food baskets are provided to the PwTB to meet any additional expenditure for nutrition or health care access, thus preventing catastrophic costs [28]. However, PwTB still incur significant OOPE [19, 29] and are at high risk of experiencing catastrophic costs [11, 30].

Available evidence on cost of TB care in India stems from studies conducted within small geographical regions and before the implementation of some of the existing support schemes [11, 19, 31, 32]. In this context, we aim to estimate the costs incurred by the PwTB in India, the proportion of households experiencing catastrophic costs due to TB, and the equity in distribution of catastrophic costs across income quintiles. We also determine the factors associated with incurring high costs and experiencing catastrophic costs due to TB diagnosis and treatment.

## Methods

### Study design

We employed a cross-sectional study design using: (i) primary data collected through interviews of PwTB, and (ii) secondary data on PwTB from *Ni-kshay* portal, the case-based real time data management system in NTEP.

### Study setting

India is administratively divided into 28 States and 8 Union Territories, which are further divided into administrative units called districts. India has a gross income per capita of US$2389 [33]. About 40.3% of the population have some health insurance coverage [34]. India is a high TB burden country with 2.4 million cases notified in 2022, of whom 30.3% were from the private sector [35]. Under the NTEP, the district TB centers monitor the program implementation through a network of sub-district level Tuberculosis Units (TUs) which oversee the first point of contact for the community with the program, namely the Peripheral Health Institutions (PHI) from both public and private sectors.

### Study population

PwTB aged ≥ 18 years notified under the NTEP, whose TB treatment outcome had been declared between May 2022 and February 2023 were eligible to participate in the study. PwTB who refused treatment, who were wrongly diagnosed, and whose treatment outcome was not evaluated, were excluded from the study.

### Sampling and sample size

The Indian states/Union Territories were divided into three strata based on TB score (a composite score measuring NTEP performance and TB burden) as high, medium and low [36]. From each stratum, three States were selected by simple random sampling. From the nine selected states (Bihar, Delhi, Gujarat, Tamil Nadu, Telangana, Uttarakhand, Meghalaya, Odisha and Rajasthan), thirty districts were selected based on probability proportionate to size (TB notification) sampling. Two TUs were selected from each of the selected NTEP districts, and two/three PHIs from each of the selected TUs using the same sampling method. The list of all PwTB in selected PHIs was obtained, and 25 to 30 PwTB were randomly selected per PHI. Assuming that 50% of all notified PwTB experience catastrophic expenditure due to TB diagnosis and treatment [19], a minimum required sample size of around 1000 persons, allowing a 5% alpha error, design effect of two, absolute precision of 5% and 20% non-response, was calculated (Figure S1).

### Data collection

Data were collected through in-person interviews for selected PwTB or family members of deceased PwTB, which were conducted in their respective households using a structured electronic questionnaire on Open Data Kit (ODK) platform. The 'Stop TB partnership’s tool' and the 'Tuberculosis patient cost surveys: a handbook' were adapted and used for calculating the costs incurred by the PwTB during TB care [13, 37] (Box 1).

**Box 1:** Operational definitions

Calculation of out-of-pocket expenditure (OOPE) incurred by the PwTB [19]	
Direct costs	Direct costs included all OOPE incurred by PwTB during TB diagnosis and treatment. Medical expenses included OOPE on consultation or registration fees, drugs, and diagnosis. Non-medical expenses included expenditure on transport, accommodation, food, and additional food supplements costs. Reimbursement or cashless transfer received through insurance schemes were subtracted from the direct costs.
Indirect costs	Indirect costs included the income (or productivity) lost due to inability or reduced ability to work because of the TB illness. It also included the income lost due to time spent on hospital visits for diagnosis, drug collection, travel and waiting time etc.
Coping costs	Coping costs were the costs of coping mechanisms adopted by PwTB and households to meet the expenditure due to TB care. These included, but were not limited to, costs due to interest on borrowed loan, loss incurred due to sales of assets, and dissolution of savings.
Total costs	Total costs were a sum of the direct, indirect and coping costs incurred during TB diagnosis and treatment.
The costs incurred during TB diagnosis and treatment were calculated as those incurred during (1) pre-diagnosis, (2) treatment and follow-up visits, (3) hospitalization. *All costs were calculated for both the PwTB and the caregiver(s).*	
Pre-diagnosis costs	The pre-diagnosis costs included the direct and indirect costs incurred by the PwTB from the onset of first symptom in the current cascade of events that led to diagnosis of TB till the date when the diagnosis was confirmed.
Treatment and follow-up costs	The treatment and follow-up costs included the direct and indirect costs incurred by the PwTB from the initiation of treatment till declaration of the treatment outcome.
Hospitalization costs	Hospitalization costs included the direct and indirect costs incurred during every episode of hospitalization during pre-diagnosis or treatment/follow-up period.
Catastrophic costs	Total costs incurred by the PwTB during TB diagnosis and treatment, exceeding a given threshold (e.g. 20%) of the household’s annual pre-TB income [37]. Household income was calculated as a sum of income from salary and all other sources for all of the members of the household.

### Data analysis

The primary data collected through the in-person interview were merged with the secondary data from *Ni-kshay* using the episode ID (*Ni-kshay* Identifier) from the current facility notification register. All costs were calculated in terms of Indian rupees (INR) and converted into US $ using the 2023 exchange rate of US $1 = 82.57 [38]. The Shapiro–Wilk test for total cost indicated a skewed nature (*p* < 0.05). The medical and non-medical direct, indirect, and total costs of TB diagnosis and treatment, categorized by person characteristics and notification sector, were summarized using median and interquartile range (IQR). As the total costs had a skewed distribution, the factors associated with total costs incurred were determined using quantile regression at 50th percentile and presented marginal means along with 95% confidence interval (CI). The proportion of PwTB incurring catastrophic costs was calculated, and a generalized linear model with the Poisson family and log link was used to identify factors associated with incurring catastrophic costs, presented as adjusted prevalence ratio (aPR) with a 95% CI.

Concentration curve and the concentration index (with 95% CI) were used to assess the extent of equality in the distribution. Income quintiles were generated by ranking the monthly income of the household in ascending order, wherein the first quintile referred to the poorest and the fifth quintile to the richest household. The distribution of the cumulative proportion of PwTB experiencing catastrophic costs across the cumulative proportion of PwTB by income quintile was plotted to generate the concentration curve. The diagonal line in the concentration curve reflects the equal distribution of the catastrophic costs (y variable) across the income quintiles (x variable) and is called the line of equality. If the concentration curve falls below the line of equality, it suggests a pro-rich distribution of the y variable. If it falls above the line of equality, then it suggests a pro-poor distribution [39]. All statistical analyses were performed using Stata V.17.0 and R V.4.3.1.

## Results

### Socio-demographic and clinical characteristics

Of the 1407 interviewed, 865 (61.5%) were male, with a mean (SD) age of 40.8 (16.8) years. About 786 (55.9%) of the PwTB were employed, and 473 (33.6%) were the primary income earner of the household. The median(IQR) monthly pre-TB income of the PwTB was US$ 96.9 (60.6, 145.3) and that of the households was US$ 181.7 (121.1, 272.5). Over 35% (n = 517) of the PwTB were enrolled for some form of medical insurance.

Among the PwTB, 136 (9.7%) were notified from the private sector, 1088 (77.3%) had pulmonary TB, 34 (2.4%) had DRTB, and 15 (1.1%) were reactive for HIV. Overall, 258 (18.3%) PwTB were hospitalized. The number of hospitalization episodes ranged from 1 to 5, and the median (IQR) duration per episode was 7 (5, 14) days (Table 1). PwTB in our study reported a median (IQR) of 2 (1, 3) hospital visits before diagnosis. The median (IQR) duration from diagnosis to treatment outcome was 169 (167, 175) days for Drug susceptible TB (DSTB) and 196 (175, 288) days for DRTB.

**Table 1:** Overall costs of TB care by sociodemographic and clinical characteristics of PwTB, India, 2022–2023 (N = 1407)

Characteristics	n (%)	Total cost median (IQR)	Direct cost median (IQR)	Indirect cost median (IQR)
Overall	1407 (100.0)	386.1 (130.8, 876.9)	78.4 (43.3, 153.6)	279.8 (18.9, 699.4)
Age (in years)				
≥ 18 to ≤ 59	1137 (80.8)	405.5 (144.9, 883.2)	79.9 (43.6, 154.8)	287.6 (30.8, 704.6)
60	270 (19.2)	259.5 (72.4, 833.2)	71.0 (40.3, 147.8)	131.8 (7.6, 576.5)
Gender				
Male	865 (61.5)	411.4 (154.7, 833.2)	72.9 (41.6, 154.2)	288.3 (41.0, 653.5)
Female	542 (38.5)	352.7 (100.1, 900.4)	85.1 (47.6, 147.8)	223.5 (10.8, 767.4)
Education				
Illiterate	527 (37.5)	391.9 (122.2, 866.3)	64.6 (36.3, 138.1)	290.4 (14.6, 699.7)
Any formal schooling	705 (50.1)	381.8 (133.2, 848.9)	78.1 (47.5, 160.5)	257.9 (21.8, 650.1)
Any college education	175 (12.4)	396.7 (142.6, 926.2)	99.9 (62.4, 178.6)	279.8 (22.3, 839.3)
Occupation^a^				
Employed	786 (55.9)	425.2 (189.0, 778.4)	72.8 (41.5, 159.5)	302.6 (78.5, 596.2)
Economically inactive	621 (44.1)	331.9 (81.3, 928.2)	81.5 (48.2, 147.6)	155.8 (5.1, 839.3)
Monthly household income (quintiles) before TB				
1st (poorest)	262 (18.6)	342.3 (79.0, 882.4)	64.5 (33.9, 136.9)	245.4 (7.5, 581.1)
2nd	337 (24.0)	363.0 (132.8, 872)	67.8 (41.9, 141.6)	250.0 (17.0, 704.6)
3rd	257 (18.3)	413.7 (159.3, 810)	79.9 (42.3, 148.8)	262.1 (55.3, 596.2)
4th	279 (19.8)	394.5 (109.8, 800.2)	75.8 (43.6, 155.8)	279.8 (16.4, 667.2)
5th (richest)	268 (19.0)	480.6 (154.1, 974.7)	96.8 (59.6, 193.1)	288.1 (31.1, 839.3)
Unknown/missing	4 (0.3)	270.1 (162.2, 325.2)	137.8 (29.1, 238.9)	79.5 (3.0, 216.5)
Health/medical insurance				
Yes	517 (36.7)	326.6 (93.9, 815.7)	84.8 (41.9, 170.3)	187.5 (14.2, 575.5)
No	890 (63.3)	414.1 (146.4, 884.9)	74.0 (43.6, 145.3)	295.1 (28.4, 728.7)
Notifying sector				
Public	1271 (90.3)	363.4 (117.6, 835.3)	71.5 (41.4, 140.7)	261.7 (18.2, 655.1)
Private	136 (9.7)	698.7(252.2, 1092.2)	159.1 (98.6, 384.8)	367.0 (25.0, 842.6)
Type of patient				
New	1194 (84.9)	396.3 (132.8, 882.4)	79.9 (43.6, 159.1)	279.8 (19.3, 716)
PMDT	34 (2.4)	495.8 (183.2, 911.2)	87.7 (50.0, 217.6)	291.2 (36, 839.3)
Retreatment	178 (12.6)	265.3 (118.2, 708.3)	57.2 (39.0, 136.0)	176.0 (10.9, 559.5)
Unknown/missing	1 (0.1)	185.3 (185.3, 185.3)	27.7 (27.7, 27.7)	157.5 (157.5, 157.5)
Site of disease				
Extra pulmonary	313 (22.3)	402.4 (142.6, 964.5)	100.5 (57.9, 197.4)	284.3 (19.9, 757.2)
Pulmonary	1088 (77.3)	384.2 (123.3, 842)	70.5 (40.1, 143.1)	258.8 (17.5, 663.4)
Unknown/missing	6 (0.4)	278.6 (185.3, 701.2)	79.6 (27.7, 168.3)	176.2 (125.2, 610.8)
Drug type				
DSTB	1372 (97.5)	385.3 (128.3, 875)	78.2 (42.9, 153.4)	279.8 (18.2, 699.4)
DRTB	34 (2.4)	495.8 (183.2, 911.2)	87.7 (50.0, 217.6)	291.2 (36.0, 839.3)
Unknown/missing	1 (0.1)	185.3 (185.3, 185.3)	27.7 (27.7, 27.7)	157.5 (157.5, 157.5)
HIV				
Reactive	15 (1.1)	529.2 (103.2, 1151.1)	167.1 (48.0, 286.1)	439.6 (7.6, 979.2)
Non-reactive	1366 (97.1)	386.7 (128.4, 876.9)	77.9 (42.9, 152.6)	279.8 (18.2, 699.4)
Unknown/missing	26 (1.8)	359.7 (181.4, 752.7)	89.0 (52.3, 127.2)	279.8 (126.2, 599.3)
Diabetes				
Diabetic	130 (9.2)	575.3 (194.6, 967)	96.3 (55.5, 186.4)	366.8 (56.8, 839.3)
Non-diabetic	1221 (86.8)	369.3 (123.6, 866.3)	76.5 (42.8, 149.4)	255.6 (17.0, 667.2)
Unknown/missing	56 (4.0)	412.6 (185.8, 730.5)	66.2 (38.2, 128.0)	330.1 (132.8, 533)
Hospitalization				
Yes	258 (18.3)	882.3 (408, 1554.1)	307.7 (149.6, 591.3)	477.6 (178.1, 921.9)
No	1149 (81.7)	317.5 (98.7, 715.4)	62.4 (39.0, 112.1)	218.3 (12.6, 590.2)
Treatment outcome				
Favourable	1309 (93.0)	387.2 (130.8, 878.2)	77.9 (43.3, 149.6)	279.8 (18.2, 704.6)
Unfavourable	98 (7.0)	356.9 (136.6, 744.6)	89.0 (43.6, 221.0)	211.1 (32.6, 535)

Costs mentioned are for all PwTB in a given category irrespective of whether they incurred the cost or not

Abbreviations: PMDT, Programmatic Management of Drug-resistant Tuberculosis; DSTB, Drug sensitive Tuberculosis; DRTB, Drug resistant Tuberculosis; HIV, Human Immunodeficiency Virus

^a^Employed (Regular employee government/Regular employee private/Temporary employee (government and private)/Skilled worker/Daily wage earner/ Business/farm/shop); Economically inactive (Unemployed/Homemaker/Retired/Pensioner/Student)

### Proportion incurring costs for TB care

Almost all (n = 1398, 99.4%) PwTB had incurred some costs due to TB diagnosis and treatment. Direct costs were incurred by 1386 (98.5%) PwTB, while indirect costs were incurred by 1307 (92.9%) PwTB. Overall, 1361 (96.7%) PwTB had incurred costs during the pre-diagnosis period, 1031 (73.3%) during treatment and follow-up, and 257 (18.3%) during hospitalization. Around 12% (n = 165) PwTB incurred coping costs (Table 2).

**Table 2:** Total, direct and indirect costs (US$) among PwTB stratified by notifying sector, India, 2022–23 (N = 1407)

Variables	Overall (N = 1407)			Public (n = 1271)			Private (n = 136)		
	Costs among all patients median (IQR)	Incurred the cost n (%)	Among those who incurred the cost median (IQR)	Among all patients median (IQR)	Incurred the cost n (%)	Among those who incurred the cost median (IQR)	Among all patient median (IQR)	Incurred the cost n (%)	Among those who incurred the cost median (IQR)
Total cost	386.1 (130.8, 876.9)	1398 (99.4)	392.7 (134.5, 878)	363.4 (117.6, 835.3)	1263 (99.4)	367.1 (122.4, 841.7)	698.7 (252.2, 1092.2)	135 (99.3)	700.2 (255.6, 1097.3)
Pre-diagnosis	18.2 (5.2, 54.2)	1361 (96.7)	19.7 (6, 56.5)	15.3 (4.8, 47.4)	1226 (96.5)	16.8 (5.4, 49.7)	63.2 (27.2, 95)	135 (99.3)	63.7 (27.4, 95.8)
During treatment	7.7 (0, 21.1)	1031 (73.3)	13.7 (6.3, 29.1)	7.3 (0, 18.8)	908 (71.4)	12.9 (6, 25.6)	27.4 (8.6, 156.2)	123 (90.4)	31.9 (12.1, 195.1)
Hospitalization	0 (0, 0)	257 (18.3)	238.8 (107.4, 650.3)	0 (0, 0)	231 (18.2)	224.1 (106.6, 620.1)	0 (0, 0)	26 (19.1)	277.6 (110.8, 1057.3)
Total direct cost	78.4 (43.3, 153.6)	1386 (98.5)	79.6 (44.4, 155.8)	71.5 (41.4, 140.7)	1252 (98.5)	72.7 (42.4, 142)	159.1 (98.6, 384.8)	134 (98.5)	159.3 (102.9, 397.8)
Pre-diagnosis	12.1 (2.3, 47.4)	1296 (92.1)	16 (3.2, 51.9)	9.4 (1.9, 40)	1162 (91.4)	12.6 (2.9, 46)	57.8 (24.2, 88.5)	134 (98.5)	59.5 (24.3, 88.7)
Medical cost	2.4 (0, 36.3)	839 (59.6)	27.9 (6.7, 66.3)	0.4 (0, 29.7)	713 (56.1)	24.2 (5.2, 59.1)	48.1 (20, 76.3)	126 (92.6)	50.9 (24.2, 79.9)
Non-medical cost	3.4 (1.0, 9.7)	1258 (89.4)	4.4 (1.6, 10.9)	3.2 (1.0, 9.7)	1129 (88.8)	4.2 (1.5, 10.9)	5.3 (1.6, 9.8)	129 (94.9)	5.6 (1.9, 10.1)
During treatment	2.9 (0, 10.9)	861 (61.2)	7.7 (3.8, 18.2)	2.1 (0, 8.3)	743 (58.5)	7.3 (3.4, 14.5)	19.2 (5.8, 137.6)	118 (86.8)	26.3 (11.5, 174.1)
Medical cost	0 (0, 0)	113 (8.0)	58.1 (6.1, 185.3)	0 (0, 0)	45 (3.5)	1.2 (0.6, 36.3)	3 (0, 125.3)	68 (50.0)	125.3 (45.4, 243.4)
Non- medical cost	2.7 (0, 9.0)	855 (60.8)	7.3 (3.6, 14.5)	1.9 (0, 7.7)	739 (58.1)	7.2 (3.4, 14.5)	8.7 (2.0, 19.3)	116 (85.3)	11.5 (4.4, 21.8)
Hospitalization	0 (0, 0)	253 (18.0)	191.3 (72.7, 423.9)	0 (0, 0)	227 (17.9)	181.7 (69.0, 423.2)	0 (0, 0)	26 (19.1)	228.7 (110.8, 702.4)
Nutrition	36.3 (24.2, 48.4)	1290 (91.7)	36.3 (24.2, 48.4)	36.3 (24.2, 50.9)	1171 (92.1)	36.3 (24.2, 50.9)	36.3 (24.2, 48.4)	119 (87.5)	36.3 (24.2, 48.4)
TOTAL INDIRECT COST	279.8 (18.9, 699.4)	1307 (92.9)	297.8 (52.9, 728.3)	261.7 (18.2, 655.1)	1180 (92.8)	291 (52.1, 715.6)	367 (25, 842.6)	127 (93.4)	431.7 (63.8, 845.9)
Loss of wages	214.9 (0, 573.0)	939 (66.7)	429.7 (214.9, 839.3)	214.9 (0, 573)	847 (66.6)	419.6 (214.9, 839.3)	292.5 (0, 839.3)	92 (67.6)	477.5 (283.1, 839.3)
Pre-diagnosis	3.8 (0.9, 7.9)	1074 (76.3)	4.9 (2.5, 9.8)	3.6 (0.9, 8.2)	969 (76.2)	5.1 (2.5, 10.1)	3.8 (1.0, 6.6)	105 (77.2)	4.4 (2.8, 7.6)
During treatment	1.9 (0, 8.9)	802 (57.0)	7.6 (3.7, 15.1)	1.7 (0, 8.5)	713 (56.1)	7.6 (3.8, 14.8)	2.7 (0, 12.1)	89 (65.4)	9.1 (3, 22.7)
Hospitalization	40.4 (14.5, 92.7)	210 (14.9)	57.2 (28.2, 122.6)	40.4 (11.2, 92.3)	187 (14.7)	56.3 (28.3, 122.6)	40.9 (20.2, 101.7)	23 (16.9)	60.6 (25.4, 151.4)
Coping cost	0 (0, 0)	165 (11.7)	87.2 (38.8, 232.5)	0 (0, 0)	137 (10.8)	87.2 (36.3, 242.2)	0 (0, 0)	28 (20.6)	96.9 (59.3, 196.2)

### Total costs of TB care

The median (IQR) total costs experienced by the PwTB during TB diagnosis and treatment was US$ 386.1 (130.8, 876.9), of which indirect costs were US$ 279.8 (18.9, 699.4) and direct costs were US$ 78.4 (43.3, 153.6). Overall, PwTB aged ≥ 18 to ≤ 59 years spent US$ 405.5 (144.9, 883.2), whereas those aged > 60 years spent US$259.5 (72.4, 833.2). PwTB who were hospitalized spent US$ 882.3 (408, 1554.1) compared to 317.5 (98.7, 715.4) spent by those who were never hospitalized. PwTB with DRTB spent US$ 495.8 (183.2, 911.2), those reactive for HIV spent US$ 529.2 (103.2, 1151.1), and those with diabetes mellitus spent US$ 575.3 (194.6, 967) (Table 1).

PwTB spent US$19.7 (6, 56.5) during the pre-diagnosis period, US$ 13.7 (6.3, 29.1) while on treatment, compared to US$ 238.8 (107.4, 650.3) during hospitalization (Table 2). Of the total costs, PwTB incurred a median (IQR) of US$ 370 (121.6, 823.8) on themselves and US$ 11.1 (3.6, 42.4) on caregiver(s) during TB diagnosis and treatment (Table S1).

Overall, indirect costs accounted for 66% of the total costs. Loss of wages (US$ 429.7; 214.9, 839.3) accounted for 83.4% of the total indirect costs. Direct medical costs accounted for 42.9% of the total direct costs, of which 56.4% was due to hospitalization (US$ 191.3; 72.7, 423.9) (Figure S2).

PwTB notified in the private sector incurred US$ 700.2 (255.6, 1097.3) as total costs and those in the public sector incurred US$ 367.1 (122.4, 841.7). Pre-diagnosis costs US$ 63.7 (27.4, 95.8) and hospitalization costs in the private sector US$ 277.6 (110.8, 1057.3) were higher than the respective costs in the public sector (Prediagnosis: US$ 16.8 (5.4, 49.7), Hospitalization: US$ 224.1 (106.6, 620.1) (Table 2)). The costs incurred in the public and private sectors by socio-demographic and clinical characteristics of PwTB are summarized in Table S2.

### Factors associated with total costs of TB care

PwTB in the age group ≥ 18 to ≤ 59 years (US$ 446.1; 95% CI 370.4, 521.9), who had no health insurance (US$ 464.3; 95% CI 386.7, 541.7) or were hospitalized (US$ 900.4; 95% CI 700.2, 1100.6) experienced significantly (*p* < 0.05) higher costs, compared to their counterparts (Table 3).

**Table 3:** Factors associated with total costs (US$) incurred by the PwTB during TB care, India, 2022–2023 (N = 1398)

Characteristics	n (%)	Total cost median (IQR)	Adjusted coefficients (95%CI)	Marginal means (95% CI)	*p* value
Overall	1398 (100)	392.7 (134.5, 878.0)			
Age (in years)					
≥ 18 to ≤ 59	1134 (81.1)	406.2 (145.3, 884.9)	108.55 (41.97, 175.13)	446.08 (370.37, 521.80)	0.001
≥ 60	264 (18.9)	261.6 (79.6, 838.8)	Reference	337.54 (280.46, 394.61)	
Gender					
Male	861 (61.6)	413.7 (160.0, 835.3)	36.00 (− 39.70, 111.71)	439.42 (354.14, 524.71)	0.351
Female	537 (38.4)	354.8 (104.1, 906.4)	Reference	403.42 (341.75, 465.09)	
Education					
Illiterate	518 (37.1)	399.2 (133.7, 874.9)	–	–	–
Any formal schooling	705 (50.4)	381.8 (133.2, 848.9)	–	–	–
Any college education	175 (12.5)	396.7 (142.6, 926.2)	–	–	–
Occupation^a^					
Employed	785 (56.1)	425.7 (189.7, 778.4)	Reference	449.77 (395.46, 504.07)	
Economically inactive	613 (43.9)	336.9 (84.8, 930.0)	− 55.13 (− 179.21, 68.95)	394.64 (270.86, 518.42)	0.384
Monthly household income before TB (quintiles)					
1st (poorest)	259 (18.5)	347.3 (82.9, 899.8)	− 81.72 (− 202.45, 39.01)	386.30 (286.37, 486.24)	0.184
2nd	336 (24)	367.8 (133.0, 874.4)	− 71.41 (− 169.02, 26.19)	396.61 (299.59, 493.63)	0.151
3rd	254 (18.2)	427.8 (170.1, 815.7)	− 30.11 (− 139.95, 79.73)	437.92 (341.67, 534.16)	0.591
4th	277 (19.8)	395.8 (117.6, 800.2)	− 22.84 (− 129.43, 83.75)	445.19 (349.77, 540.61)	0.674
5th (richest)	268 (19.2)	480.6 (154.1, 974.7)	Reference	468.03 (387.41, 548.64)	
Unknown/Missing	4 (0.3)	270.1 (162.2, 325.2)	–	–	–
Health/medical insurance scheme					
Yes	514 (36.8)	328.1 (101.3, 819.2)	− 105.01 (− 206.75, − 3.27)	359.16 (265.89, 452.43)	0.043
No	884 (63.2)	420.3 (155.9, 887.5)	Reference	464.17 (386.73, 541.62)	
Recipients of NPY					
Yes	1250 (89.4)	393.2 (134.5, 881.4)	–	–	–
No	148 (10.6)	389.0 (117.2, 826.6)	–	–	–
Notifying sector					
Public	1263 (90.3)	367.1 (122.4, 841.7)	Reference	398.68 (336.57, 460.78)	
Private	135 (9.7)	700.2 (255.6, 1097.3)	278.05 (− 66.69, 622.79)	676.72 (332.40, 1021.05)	0.114
Site of disease					
Extra Pulmonary	313 (22.4)	402.4 (142.6, 964.5)	–	–	–
Pulmonary	1079 (77.2)	391.9 (130.8, 845.6)	–	–	–
Unknown/Missing	6 (0.4)	278.6 (185.3, 701.2)	–	–	–
Drug type^b^					
DSTB	1363 (97.5)	391.9 (133.7, 877.0)	–	–	–
DRTB	34 (2.4)	495.8 (183.2, 911.2)	–	–	–
Unknown/Missing	1 (0.1)	185.3 (185.3, 185.3)	–	–	–
HIV					
Reactive	15 (1.1)	529.2 (103.2, 1151.1)	–	–	–
Non–Reactive	1358 (97.1)	393.2 (133.7, 878.0)	–	–	–
Unknown/Missing	25 (1.8)	364.5 (185.3, 752.7)	–	–	–
Diabetes					
Diabetic	129 (9.2)	577.9 (195.1, 967.0)	57.51 (− 63.63, 178.66)	474.54 (360.58, 588.51)	0.352
Non-Diabetic	1214 (86.8)	373.0 (125.5, 866.6)	Reference	417.03 (344.55, 489.51)	
Unknown/Missing	55 (3.9)	428.8 (186.4, 752.7)	82.40 (− 58.04, 222.85)	499.44 (358.71, 640.17)	0.250
Hospitalization					
Yes	258 (18.5)	882.3 (408.0, 1554.1)	518.60 (379.09, 784.10)	900.39 (700.20, 1100.59)	< 0.001
No	1140 (81.5)	323.4 (101.5, 719.4)	Reference	318.80 (254.58, 383.01)	
Treatment outcome					
Favourable	1302 (93.1)	392.7 (134.1, 881.4)	–	–	–
Unfavourable	96 (6.9)	386.7 (138.1, 759.6)	–	–	–

For marginal means, *p* value of 0.2 is considered for statistical significance

*DSTB* drug sensitive tuberculosis; *DRTB* drug resistant tuberculosis; *HIV* human immunodeficiency virus

^a^Employed (Regular employee government/Regular employee private/Temporary employee (government and private)/Skilled worker/Daily wage earner/ Business/farm/shop); Economically inactive (Unemployed/Homemaker/Retired/Pensioner/Student)

^b^n is very low in the in unknown/missing category, so it was not considered for unadjusted analysis

### Catastrophic costs for TB care

**Fig. 1:**
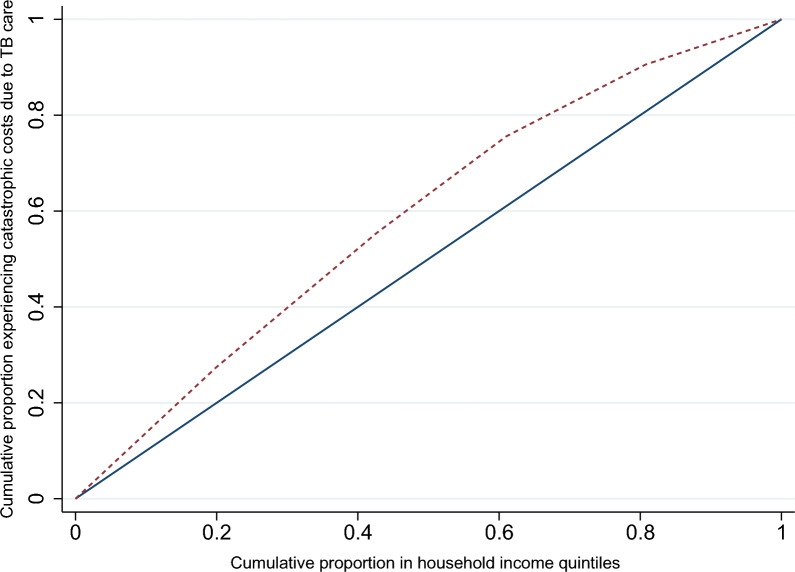
Concentration curve showing distribution of catastrophic TB costs experienced by the PwTB, India, 2022 (N = 1407). DC-Direct cost, TC- Total cost, AHI- Annual Household Income

Catastrophic costs for TB care were experienced by 634 (45.5%) of the households with PwTB. Concentration curve followed a pro-poor distribution with greater concentration of catastrophic costs in the poorer quintiles (*p* < 0.001) (Fig. 1).

PwTB notified under private sector (aPR = 1.4; 95% CI 1.1, 1.8), those who were hospitalized (aPR = 1.9 95% CI 1.6, 2.2) and those from poorest quintile (aPR = 3.2; 95% CI 2.4, 4.4) were at higher risk of experiencing catastrophic costs (Table 4).

**Table 4:** Factors associated with catastrophic costs incurred by the PwTB during TB care, India, 2022–2023 (N = 1394^a^)

Characteristics	n	Number incurring catastrophic costs n(%)	Unadjusted PR (95% CI)	Adjusted PR (95% CI)	*p* value
Age (in years)*					
≥ 18 to ≤ 59	1131	532 (47.0)	1.21 (0.99, 1.49)	1.23 (0.99, 1.54)	0.065
≥ 60	263	102 (38.8)	1	1	
Gender*					
Male	859	425 (49.5)	1.27 (1.10, 1.46)	1.13 (0.92, 1.37)	0.245
Female	535	209 (39.1)	1	1	
Education*					
Illiterate	516	265 (51.4)	1.38 (1.11, 1.70)	1.01 (0.76, 1.35)	0.929
Any formal schooling	704	304 (43.2)	1.16 (0.92, 1.45)	0.89 (0.67, 1.17)	0.394
Any College education	174	65 (37.4)	1	1	
Occupation^b^*					
Employed	783	395 (50.4)	1	1	
Economically inactive	611	239 (39.1)	0.78 (0.65, 0.92)	0.84 (0.69, 1.02)	0.073
Monthly household income before TB (quintiles) *					
1st (poorest)	259	171 (66.0)	3.05 (2.32,4.01)	3.21 (2.36, 4.36)	< 0.001
2nd	336	184 (54.8)	2.53 (1.97, 3.25)	2.56 (1.89, 3.45)	< 0.001
3rd	254	124 (48.8)	2.26 (1.79, 2.85)	2.27 (1.66, 3.12)	< 0.001
4th	277	97 (35.0)	1.62 (1.25, 2.10)	1.64 (1.18, 2.28)	0.003
5th (richest)	268	58 (21.6)	1	1	
Health/medical insurance scheme*					
Yes	512	212 (41.4)	0.87 (0.70, 1.07)	0.85 (0.72, 1.00)	0.050
No	882	422 (47.8)	1	1	
Recipients of NPY					
Yes	1247	573 (46.0)	1.11 (0.87, 1.41)	–	–
No	147	61 (41.5)	1	–	–
Notifying sector*					
Public	1259	551 (43.8)	1	1	
Private	135	83 (61.5)	1.41 (0.99, 1.99)	1.39 (1.10, 1.76)	0.005
Site of disease					
Extra Pulmonary	312	140 (44.9)	0.98 (0.82, 1.18)	–	–
Pulmonary	1076	491 (45.6)	1	–	–
Missing	6	3 (50.0)	–	–	–
Drug type					
DSTB	1359	619 (45.6)	1.03 (0.67, 1.58)	–	–
DRTB	34	15 (44.1)	1	–	–
Missing	1	–	–	–	–
HIV					
Reactive	15	8 (53.3)	1.18 (0.71, 1.95)	–	–
Non-Reactive	1354	614 (45.4)	1	–	–
Unknown/Missing	25	12 (48.0)	1.06 (0.75, 1.50)	–	–
Diabetes					
Diabetic	129	64 (49.6)	1.11 (0.92, 1.33)	–	–
Non-Diabetic	1210	541 (44.7)	1	–	–
Unknown/Missing	55	29 (52.7)	1.18 (0.87, 1.59)	–	–
Hospitalization*					
Yes	256	178 (69.5)	1.74 (1.49, 2.02)	1.87 (1.57, 2.22)	< 0.001
No	1138	456 (40.1)	1	1	
Treatment outcome					
Favourable	1298	590 (45.5)	1	–	–
Unfavourable	96	44 (45.8)	1.01 (0.81, 1.26)	–	–

For adjusted PR, *p* value of 0.2 is considered for statistical significance;*factors whose association with catastrophic Tb costs has *p* < 0.2, 1—reference category

*DSTB* drug sensitive tuberculosis; *DRTB* drug resistant tuberculosis; *HIV* human immunodeficiency virus

^a^Household income was not available for 4 PwTB and 9 had not incurred any cost during pre-diagnosis and treatment

^b^Employed (Regular employee government/Regular employee private/Temporary employee (government and private)/Skilled worker/Daily wage earner/Business/farm/shop); Economically inactive (Unemployed/Homemaker/Retired/Pensioner/Student)

## Discussion

In this first nationally representative, cross-sectional study on cost of TB care in India, almost all PwTB had experienced some costs towards TB care. The median total cost was US$ 386.1. Hospitalization was the major driver of direct and indirect costs. Indirect costs due to loss of wages or productivity contributed more to the total costs compared to direct costs. Forty five percent of the households experienced catastrophic costs, especially those who were from poorer income quintiles, had experienced hospitalization or had sought care in the private sector.

Compared to our findings, studies conducted using the WHO tool in neighbouring LMICs like China (US$2389.5) [25], Thailand (US$903) [40], and Myanmar (US$759) [17] have reported high costs for TB diagnosis and care. Indirect costs contributed to two-thirds of the total costs.Though TB diagnosis and treatment is free of cost under the NTEP, PwTB incur loss of wages and experience loss of productivity both due to periods of absence from paid work, visits to the health facilities for diagnosis, drug collection or follow-up investigations. Some PwTB are also forced to take up a lesser paying job due to TB symptoms or sequelae adding to indirect costs of the disease. Since a sizeable Indian working population is employed in unorganized sector [41] with no benefits such as paid sick leave or employer offered insurance, the indirect cost of TB is higher in India [11]. Understandably, in our analysis, the economically productive age group of 18 to 59 years experienced higher total costs compared to the older PwTB, the difference mostly driven by the indirect costs.

Direct costs were incurred mostly before diagnosis or during hospitalization for TB diagnosis or treatment. A number of health facility visits are made before a person with presumptive TB is diagnosed with TB, with longer pre-diagnosis period leading to higher expenditure. On average, a person with presumptive TB in India, visits three healthcare providers before receiving a diagnosis [42]. Three-fourths of them have their first encounter in the private sector and undergo at least three investigations, including at least one radiological investigation [43], before being diagnosed with TB and starting anti-TB- treatment. Lack of awareness and access to timely TB diagnosis and treatment could also lead to longer pathways to care, contributing to the higher costs experienced by them [44].

Available evidence shows that a person with presumptive TB, whose first contact is in the private sector, was found to have more health facility visits, longer delays in diagnosis, higher costs, and greater risk of experiencing catastrophic TB care related costs [42, 43, 45–47]. Over 60% of people with presumptive TB approach the private sector as their first point of care. The vast and disorganized private sector, where there is minimum adherence to standard diagnostic and treatment guidelines of TB, puts PwTB at risk of experiencing significant OOPE in a country like India with suboptimal health insurance coverage and limited range of available public insurance policies [48]. Scaling up of private sector engagement focusing on both improving diagnosis and treatment outcomes has been proposed to be a cost-effective strategy for India [49].

Though tribal support scheme provides US$9.1 under the NTEP to facilitate the access to healthcare facilities, PwTB incur high costs for travel and accommodation. As it is also reported in our study, expenditure on a special diet or additional nutritional supplements has been found to contribute mainly to direct non-medical costs [50]. The NPY, a direct benefit transfer scheme under the NTEP offers US$7 monthly to all notified PwTB for their nutritional support. However, though two-thirds of the notified PwTB receive at least one benefit of NPY, they experience a significant median delay of three months after diagnosis before its receipt [51]. NTEP may focus on timely credit of NPY benefits to all PwTB while also exploring alternative methods of nutritional support through public distribution systems, cash vouchers to those especially vulnerable [50]. 

The 18.5% of the PwTB who were ever hospitalized during TB diagnosis or treatment experienced 2.5 times higher costs than those who were not hospitalized, especially if they were hospitalized in the private sector or had longer hospitalizations. Standard diagnostic and treatment guidelines, active case finding, early diagnosis, triage and differentiated care on diagnosis can reduce unnecessary hospitalizations while also improving TB treatment outcomes [31, 52].

Almost half of the households with PwTB in our study had experienced catastrophic costs. The estimates of PwTB in India experiencing catastrophic costs range from 7 to 68% [11, 19, 23, 53–57]. The lower income quintiles are at higher risk of experiencing financial catastrophe, though their actual expenditure is comparable to or lesser than that of the higher quintiles. The pro-poor distribution of catastrophic costs may also be a consequence of poor access to optimal and affordable TB diagnostic and care services among the poorer income quintiles. This is particularly of concern, as this group is more vulnerable in terms of clustering of other risk factors for TB and poorer outcomes such as low Body Mass Index (BMI) and indoor air pollution [58].

### Strengths

The cost calculation was done comprehensively using a tool adapted from the 'Stop TB partnership’s tool' for cost estimation. We have captured costs comprehensively beginning from the pre-diagnosis period to the treatment outcome. There have been no extrapolations, as actual costs incurred have been captured. The inclusion of PwTB in the study irrespective of their clinical characteristics, notifying sector and outcome, have ensured that our estimates are representative of all PwTB notified under the NTEP in India.

### Limitations

Since the costs were self-reported and captured in a cross-sectional study at the end of treatment, the recall limitation of the participants may influence the cost estimates. Objective verification of the expenditure by verification of bills, receipts or prospective recording of expenditure would have ensured more accurate capture of data. However, with a standardized, comprehensive tool and rigorous training of data collectors to use the right probes to elicit cost information, we believe that any potential bias is minimal. Due to the smaller numbers in some sub-sections of the PwTB like persons who were HIV reactive or with DR-TB, we could not comment on the costs incurred by them. Though TB may impose ongoing costs on PwTB and their households even after their treatment outcome is declared, due to the limitations imposed by our study follow-up period, we could not capture the costs incurred by patients during post treatment.

### Recommendations and implications for policy

Targeting vulnerable populations through TB-sensitive strategies that extend beyond focused TB diagnosis and treatment, like improving nutrition, reducing financial risk, and reducing TB transmission, is recommended. Standardizing diagnostic algorithms across public and private care providers, monitoring adherence to these algorithms, and incentivizing early notification of TB cases [50] may contribute significantly to reducing the number of pre-diagnosis visits, the consequent delays, hospitalization, and the costs incurred by the PwTB. Since a significant contribution to the indirect costs was due to inability to work, the focus must be on preventing severe illness due to TB and its early detection and treatment so that the productivity loss is minimal. For those in the organized sector, paid leave for a fixed period may help PwTB recuperate better without progressing to severe illness or financial catastrophe and reduced workplace transmission of TB. Timely disbursal of the NPY benefits to PwTB will help them meet the expenditure on additional nutritional demands and prevent catastrophic costs. Expanding the net of coverage of health insurance schemes, both public and private, to cover TB diagnosis, treatment and rehabilitation will encourage better health seeking and also reduce financial catastrophe in households of PwTB. Future costing exercises may also calculate the costs incurred by patients after declaration of their treatment outcome, at least for a period of two years to capture costs due to physical, social and economic sequelae of TB.

## Conclusions

Despite free TB diagnostic and treatment services under the NTEP, PwTB continue to incur high costs, mostly driven by indirect costs due to lost productivity. Nearly half of the PwTB incur catastrophic costs, which are disproportionately concentrated among poorer income quintiles. Seeking treatment in the private sector and hospitalization increase the risk of incurring catastrophic costs.

## Supplementary Information

Additional file 1.

## Data Availability

The datasets used and analyzed during the current study are available from the corresponding author on reasonable request.

## Abbreviations

aPR: Adjusted prevalence ratio
BMI: Body mass index
CI: Confidence interval
DRTB: Drug resistant TB
ICMR-NIE: Indian Council of Medical Research-National Institute of Epidemiology
IHEC: Institutional Human Ethics Committee
IQR: Interquartile range
JSI: John Snow Research & Training Institute Inc
LMIC: Low and middle-income countries
NPY: *Ni-kshay Poshan Yojana*
NTEP: National TB Elimination Programme
ODK: Open data kit
OOPE: Out-of-pocket expenditure
PHI: Peripheral Health Institutions
PwTB: Persons with TB
SD: Standard deviation
TB: Tuberculosis
TIFA: Tuberculosis Implementation Framework Agreement
TU: Tuberculosis units
USAID: United States Agency for International Development
