# Supplementary material for: Cost of TB care and equity in distribution of catastrophic TB care costs across income quintiles in India
Source: Glob Health Res Policy. 2024 Dec 9;9:51. doi: 10.1186/s41256-024-00392-9 (PMC11626761; doi:10.1186/s41256-024-00392-9)
Supplement: Supplementary file 1 — Additional file 1. [file 41256_2024_392_MOESM1_ESM.docx]

**Supplementary material**

Table S1. **Patient costs and guardian costs (US$) incurred during TB care stratified by notifying sector, India, 2022-23 (N=1407)**

| **Variables** | **Overall (N)** | | |
| --- | --- | --- | --- |
|  | **Among all patients**  **Median (IQR)** | **n (%)** | **Among those who incurred the cost**  **Median (IQR)** |
| **Total cost** | 386·1 (130·8, 876·9) | 1398 (99·4) | 392·7 (134·5, 878) |
| **Patient cost** | 363·6 (117·8, 820·5) | 1398 (99·4) | 370 (121·6, 823·8) |
| **Total direct cost** | 73·2 (42·3, 144·9) | 1386 (98·5) | 74·2 (43·6, 145·9) |
| **Pre-diagnosis** | 10·9 (1·5, 45·1) | 1295 (92·0) | 14·8 (2·4, 50·8) |
| Medical cost | 2·4 (0, 36·3) | 839 (59·6) | 27·9 (6·7, 66·4) |
| Non-medical cost | 2·4 (0·7, 7·3) | 1255 (89·2) | 3·2 (1·1, 9) |
| **During treatment** | 2·4 (0, 8) | 859 (61·1) | 6·7 (2·9, 14·5) |
| Medical cost | 0 (0, 0) | 113 (8·0) | 58·1 (6·1, 185·3) |
| Non-medical cost | 2·2 (0, 7·3) | 853 (60·6) | 5·8 (2·9, 12) |
| Hospitalization | 122·9 (45·8, 363·3) | 251 (17·8) | 125·1 (48·8, 363·9) |
| Nutrition | 36·3 (24·2, 48·4) | 1290 (91·7) | 36·3 (24·2, 48·4) |
| **Total Indirect costs** | 246·6 (10·2, 610·1) | 1206 (85·7) | 325·9 (90·1, 721·9) |
| Inability to work | 209·8 (0, 573) | 935 (66·5) | 419·6 (214·9, 839·3) |
| Pre-diagnosis | 0.8 (0, 4.0) | 751 (53·4) | 3·8 (1·9, 7·1) |
| During treatment | 0 (0, 5·7) | 625 (44·4) | 7·4 (3·4, 15·1) |
| Hospitalization | 9·8 (0, 40·4) | 141 (10·0) | 36·3 (17·7, 68·1) |
| Total coping cost | 0 (0, 0) | 165 (11·7) | 87·2 (38·8, 232·5) |
| **Guardian cost** | 3·6 (0, 19·2) | 938 (66·7) | 11·1 (3·6, 42·4) |
| **Total direct costs** | 0 (0, 6·1) | 657 (46·7) | 6·8 (2·4, 24·2) |
| Pre-diagnosis | 0 (0,1.2) | 520 (37·0) | 2·2 (1·1, 4·8) |
| During treatment | 0 (0, 0) | 297 (21·1) | 3·6 (1·8, 7·3) |
| Hospitalization | 0 (0, 0) | 218 (15·5) | 32·1 (12·7, 72·7) |
| **Total Indirect costs** | 0 (0, 9.5) | 652 (46·3) | 11·9 (3·8, 42·3) |
| Loss of wages | 0 (0,0) | 82 (5·8) | 109 (54·5, 218) |
| Pre-diagnosis | 0 (0, 3.2) | 594 (42·2) | 4·2 (1·9, 8·3) |
| During treatment | 0 (0,0) | 302 (21·5) | 4·5 (2·3, 9·8) |
| Hospitalization | 9·7 (0, 50·9) | 145 (10·3) | 43·6 (18·2, 141·2) |

Table S2**. Overall costs (US$) of TB care by socio-demographic and clinical characteristics of persons with TB stratified by notifying sector, India, 2022-23 (N=1407)**

| **Characteristics** | **Public (n=1271)** | | | | **Private (n=136)** | | | |
| --- | --- | --- | --- | --- | --- | --- | --- | --- |
|  | **N (%)** | **Total cost**  **Median (IQR)** | **Direct cost**  **Median (IQR)** | **Indirect cost**  **Median (IQR)** | **N (%)** | **Total cost**  **Median (IQR)** | **Direct cost**  **Median (IQR)** | **Indirect cost**  **Median (IQR)** |
| **Overall** | 1271 (100.0) | 363.4 (117.6, 835.3) | 71.5 (41.4, 140.7) | 261.7 (18.2, 655.1) | 136 (100.0) | 698.7 (252.2, 1092.2) | 159.1 (98.6, 384.8) | 367 (25, 842.6) |
| **Age (in Years)** |  |  |  |  |  |  |  |  |
| ≥18 to ≤59 | 1033 (81.3) | 393.5 (137.1, 859.5) | 73.3 (42.6, 142.9) | 284.8 (30.8, 684.3) | 104 (76.5) | 684.2 (244.2, 1146.7) | 159.8 (94.8, 403.3) | 425.4 (30.1, 842.6) |
| ≥60 | 238 (18.7) | 226.3 (62.7, 769.5) | 63.3 (38.4, 126.9) | 99.3 (6.7, 547.6) | 32 (23.5) | 828.0 (255.2,1009.2) | 144.9 (101.9, 277.3) | 309.2 (20.4, 842.8) |
| **Gender** |  |  |  |  |  |  |  |  |
| Male | 780 (61.4) | 396.3 (142.5, 802.7) | 66 (39.5, 137.6) | 288 (45.1, 649.7) | 85 (62.5) | 592.8 (255.6, 1114.3) | 171.5 (100.9, 371.8) | 328 (32.2, 730.2) |
| Female | 491 (38.6) | 328.9 (93.8, 876.9) | 80.1 (45.1, 142.9) | 209.8 (10.2, 701.6) | 51 (37.5) | 878.3 (232.8, 1018.7) | 140.9 (96.3, 397.8) | 532.7 (18.2, 846.9) |
| **Education** |  |  |  |  |  |  |  |  |
| Cannot read or write | 465 (36.6) | 339.8 (103.2, 762.3) | 56.3 (33.9, 124.5) | 258 (13, 651.4) | 62 (45.6) | 885.2 (403.2, 1100.9) | 140.5 (96.3, 313.7) | 481.5 (90.9, 843.7) |
| Any formal schooling | 653 (51.4) | 370 (123.2, 823.8) | 73.8 (45.9, 145.3) | 257.9 (25.4, 633.6) | 52 (38.2) | 606.0 (199.5, 1168.7) | 182.2 (104.2, 436.1) | 256.7 (15.7, 845.6) |
| Any Graduates | 153 (12.0) | 396.7 (135.8, 926.2) | 93.5 (60.7, 164) | 279.8 (22.3, 839.3) | 22 (16.2) | 426.3 (203.6, 919.2) | 160.4 (100.9, 247.5) | 263.1 (32.2, 721.9) |
| **Occupation ^a^** |  |  |  |  |  |  |  |  |
| Employed | 712 (56.0) | 408.7 (184.6, 756) | 65.2 (39.8, 139.4) | 303.3 (81.7, 597.4) | -  74 (54.4) | 579.9 (255.6, 1107.7) | 210.2 (122.1, 430.2) | 284.6 (37.8, 564.6) |

| **Characteristics** | **Public (n=1271)** | | | | **Private (n=136)** | | | |
| --- | --- | --- | --- | --- | --- | --- | --- | --- |
|  | **N (%)** | **Total cost**  **Median (IQR)** | **Direct cost**  **Median (IQR)** | **Indirect cost**  **Median (IQR)** | **N (%)** | **Total cost**  **Median (IQR)** | **Direct cost**  **Median (IQR)** | **Indirect cost**  **Median (IQR)** |
| Economically inactive | 559 (44.0) | 290.7 (74.2, 911.2) | 77.3 (45.4, 141.5) | 147.1 (4.5, 839.3) | 62 (45.6) | 885.1 (210.1, 1033.6) | 133.9 (83.9, 312.1) | 699.6 (12.1, 907.4) |
| **Monthly household Income before TB (quintiles)** |  |  |  |  |  |  |  |  |
| 1st (poorest) | 234 (18.4) | 297 (71.5, 866.3) | 58 (32.1, 124.1) | 215.3 (6, 578.5) | 28 (20.6) | 637.9 (427.6, 960.3) | 133.7 (108, 261.8) | 451.2 (316.2, 840.6) |
| 2nd | 312 (24.6) | 350.8 (124.8, 850.8) | 66.2 (40.3, 130.5) | 245 (15.4, 699.4) | 25 (18.4) | 828.7 (248.7, 1268.0) | 129 (63, 364.5) | 335.2 (31.5, 907.4) |
| 3rd | 230 (18.1) | 368.4 (145, 728.1) | 70.1 (40.6, 140.7) | 259.8 (49.5, 585) | 27 (19.9) | 822.8 (347.7, 1107.7) | 186.7 (100.9, 437.3) | 431.7 (120.1, 847.8) |
| 4th | 249 (19.6) | 373.5 (105.8, 756.9) | 72.2 (42, 130.8) | 279.8 (17.7, 614.2) | 30 (22.1) | 552.7 (159.0, 954.3) | 159.2 (83.9, 371.8) | 243 (5, 842.6) |
| 5th (richest) | 242 (19.0) | 478.6 (139.4, 964.9) | 90.5 (55.5, 181.7) | 294.2 (33.2, 839.3) | 26 (19.1) | 757.2 (160.4, 1018.7) | 166.6 (128.8, 286.1) | 228.5 (19.3, 842.6) |
| Unknown/Missing | 4 (0.3) | 270.1 (162.2, 325.2) | 137.8 (29.1, 238.9) | 79.5 (3, 216.5) | NA | NA | NA | NA |
| **Site of disease** |  |  |  |  |  |  |  |  |
| Extra Pulmonary | 277 (21.8) | 408.9 (144.5, 957.9) | 95.9 (55.9, 181.7) | 287.7 (30.8, 745.2) | 36 (26.5) | 291.1 (132.4, 1082.5) | 134.2 (77.2, 328.4) | 64.1 (6.3, 877.8) |
| Pulmonary | 988 (77.7) | 347.3 (103.6, 785.5) | 64.3 (38.8, 128.2) | 236.6 (15.6, 645.7) | 100 (73.5) | 821.7 (381.6, 1092.2) | 161.1 (112.2, 416.4) | 431.5 (128.2, 842.3) |
| Unknown/Missing | 6 (0.5) | 278.6 (185.3, 701.2) | 79.6 (27.7, 168.3) | 176.2 (125.2, 610.8) | NA | NA | NA | NA |

| **Characteristics** | **Public (n=1271)** | | | | **Private (n=136)** | | | |
| --- | --- | --- | --- | --- | --- | --- | --- | --- |
|  | **N (%)** | **Total cost**  **Median (IQR)** | **Direct cost**  **Median (IQR)** | **Indirect cost**  **Median (IQR)** | **N (%)** | **Total cost**  **Median (IQR)** | **Direct cost**  **Median (IQR)** | **Indirect cost**  **Median (IQR)** |
| **Drug type** |  |  |  |  |  |  |  |  |
| DSTB | 1236 (97.2) | 363 (116.6, 825.1) | 70.7 (40.7, 139.8) | 258.7 (17.7, 653) | 136 (100.0) | 687.7 (252.2, 1092.2) | 159.1 (98.6, 384.8) | 367 (25, 842.6) |
| DRTB | 34 (2.7) | 495.8 (183.1, 911.2) | 87.7 (50, 217.6) | 291.2 (36, 839.3) | NA | NA | NA | NA |
| Unknown/Missing | 1 (0.1) | 185.3 (185.3, 185.3) | 27.7 (27.7, 27.7) | 157.5 (157.5, 157.5) | NA | NA | NA | NA |
| **HIV** |  |  |  |  |  |  |  |  |
| Reactive | 14 (1.1) | 504.9 (103.2, 1151.1) | 142.3 (48, 179) | 401.4 (7.6, 979.2) | 1 (0.7) | 792.8 (792.8, 792.8) | 286.1 (286.1, 286.1) | 506.7 (506.7, 506.7) |
| Non-Reactive | 1234 (97.1) | 364.6 (116.9, 841.7) | 70.7 (40.8, 139.9) | 261.5 (17.7, 656.5) | 132 (97.1) | 684.2 (242.0, 1092.2) | 158.6 (98.6, 371.5) | 352.1 (20.4, 842.6) |
| Unknown/Missing | 23 (1.8) | 317.6 (164.8, 535.3) | 72.1 (49.7, 124.5) | 204.3 (125.2, 364.9) | 3 (2.2) | 934.4 (919.2, 1564.2) | 499.6 (93.3, 642.3) | 841.2 (419.6, 921.9) |
| **Diabetes** |  |  |  |  |  |  |  |  |
| Diabetic | 121 (9.5) | 572.7 (191.1, 958.1) | 90.8 (52.7, 178.5) | 370.9 (52.9, 839.3) | 9 (6.6) | 605.6 (499.0, 1230.5) | 247.5 (177.7, 517.9) | 342.1 (251.5, 483.5) |
| Non-Diabetic | 1101 (86.6) | 351.3 (113.8, 815.7) | 70.2 (40.3, 138.9) | 246.8 (17, 649.1) | 120 (88.2) | 676.0 (221.5, 1026.1) | 146.7 (90.9, 329.8) | 345 (17.8, 842.6) |
| Unknown/Missing | 49 (3.9) | 366.5 (181.4, 535.3) | 52.3 (37.3, 96.9) | 279.8 (126.2, 439.6) | 7 (5.1) | 934.4 (919.2, 1815.5) | 631.3 (397.8, 1110.3) | 705.2 (419.6, 921.9) |
| **Hospitalization** |  |  |  |  |  |  |  |  |
| Yes | 232 (18.3) | 843.5 (396.4, 1494.5) | 288.1 (144.2, 551.6) | 477.6 (172.3, 899) | 26 (19.1) | 1267.7 (767.2, 1815.5) | 636.8 (249.7, 1043) | 586 (182.3, 1059.8) |
| **Characteristics** | **Public (n=1271)** | | | | **Private (n=136)** | | | |
|  | **N (%)** | **Total cost**  **Median (IQR)** | **Direct cost**  **Median (IQR)** | **Indirect cost**  **Median (IQR)** | **N (%)** | **Total cost**  **Median (IQR)** | **Direct cost**  **Median (IQR)** | **Indirect cost**  **Median (IQR)** |
| No | 1039 (81.7) | 291.1 (88.2, 673.5) | 58.6 (38, 101.1) | 215.2 (12.6, 583.3) | 110 (80.9) | 566.4 (191.2, 954.3) | 137.4 (86.8, 272.5) | 331.6 (15.1, 839.3) |
| **Treatment outcome** |  |  |  |  |  |  |  |  |
| Favourable | 1180 (92.8) | 363.2 (117.2, 844.5) | 70.6 (41, 138.1) | 267.9 (17.1, 670.4) | 129 (94.9) | 700.2 (285.1, 1033.6) | 159 (96.3, 371.8) | 371.6 (32.2, 842.6) |
| Unfavourable | 91 (7.2) | 366.5 (123.9, 730.1) | 84.4 (42.9, 194.4) | 227.5 (36, 535) | 7 (5.1) | 164.2 (143.9, 1564.2) | 159.5 (137.3, 642.3) | 11.4 (4.6, 921.9) |

Abbreviations: DSTB, Drug sensitive Tuberculosis; DRTB, Drug resistant Tuberculosis; HIV, Human Immunodeficiency Virus

**^a^**Self-employed (Business/farm/shop); Employed (Regular employee government/Regular employee private/Temporary employee (government and private)/Skilled worker/Daily wage earner); Economically inactive (Unemployed/Homemaker/Retired/Pensioner/Student)·

Figure S1. **Sampling scheme for selection of PwTB in India, 2022-23 (N=1407)**

Probability proportionate to size sampling (Case notification per lakh population)

Simple random sampling

Stratified random sampling

**1407 PwTB**

India

9 states

30 districts

61 TB units

138 PHIs

Three strata; one state per stratum

Districts: 3-4 per state

TB units: 2-3 per district

Peripheral Health Institutions: 2-3 per TB unit

PwTB whose treatment outcome declared between May to Oct 2022: 10 to 15 per PHI

9

PwTB- Person with Tuberculosis, PHI- Peripheral Health Institution

Figure S2. **Costs incurred by the PwTB during TB care, India, 2022-23**


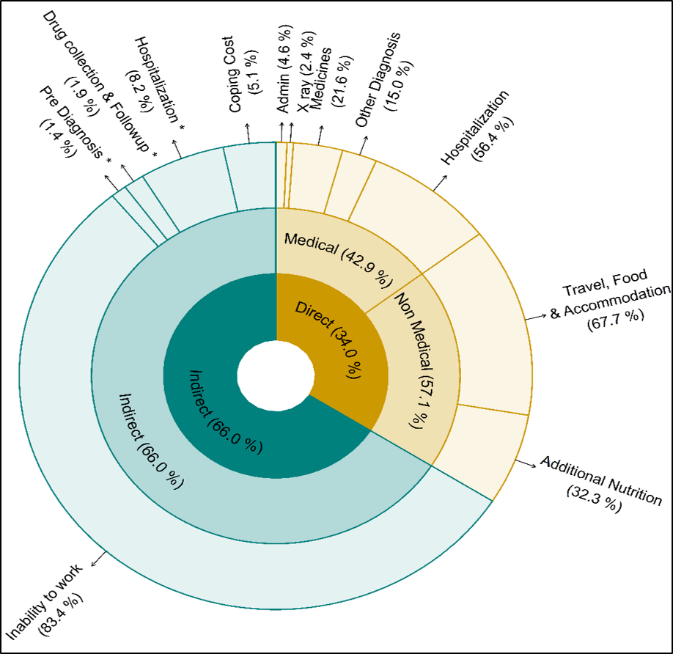


^a^Productivity loss due to travel or waiting time at the health facility
